# Diagnostic Accuracy of Isotropic FLAIR-T2* Fusion Imaging for Central Vein Sign Detection in Multiple Sclerosis: a Comparative Study at 1.5 T and 3 T

**DOI:** 10.1007/s00062-025-01531-6

**Published:** 2025-06-16

**Authors:** Yunus Emre Senturk, Ahmet Peker, Hande Ozen Atalay, Ayse Altintas, Ali Yusuf Oner

**Affiliations:** 1 Department of Radiology, Koç University Hospital, Davutpasa Street, No 4, 34010 Istanbul, Turkey; 2 Department of Neurology, Koç University Hospital, Istanbul, Turkey

**Keywords:** Multiple sclerosis, Central vein sign, 1.5 T, T2*, FLAIR

## Abstract

**Purpose:**

The central vein sign (CVS) is a promising imaging biomarker for multiple sclerosis (MS) diagnosis. While isotropic T2* at 3 T and 7 T has demonstrated high diagnostic performance, its utility at 1.5 T remains unclear. This study evaluates the performance of unenhanced FLAIR-T2* fusion at 1.5 T compared to 3 T in MS participants.

**Methods:**

This prospective observational study included 20 MS patients and 20 control subjects. Each participant underwent unenhanced isotropic Epi-T2* and isotropic FLAIR (0.8 mm voxel size) at both 1.5 T and 3 T. Subsequently, the derived isotropic T2* and FLAIR were combined to create the final FLAIR-T2* fusion in both magnetic field strengths. Two independent raters assessed the CVS status of white matter (WM) lesions using NAIMS criteria. WM lesions were classified as CVS+ or CVS-, and two methods—select-n* and CVS+ proportion—were applied. Sensitivity and specificity were computed, and CVS performance was compared across WM lesion locations.

**Results:**

Among eligible WM lesions (MS: 258; controls: 255), the mean CVS+ lesion proportion per participant was 66.9 ± 15.4% for 1.5 T FLAIR-T2* and 77.0 ± 13.6% for 3 T FLAIR-T2* (*p* < 0.01). At a 40% threshold, 1.5 T FLAIR-T2* achieved 90% sensitivity and 95% specificity, while 3 T FLAIR-T2* achieved 100% sensitivity and 95% specificity. The Select-6* method resulted in only one MS patient being misclassified at both field strengths. 3 T FLAIR-T2* detected more CVS+ lesions in deep WM (87.5% vs. 57.1%, *p* = 0.05).

**Conclusion:**

1.5 T FLAIR-T2* fusion demonstrates high performance in CVS assessment, although slightly outperformed by 3 T FLAIR-T2*. The select-6* method may enhance 1.5 T performance, supporting its feasibility for CVS evaluation.

**Supplementary Information:**

The online version of this article (10.1007/s00062-025-01531-6) contains supplementary material, which is available to authorized users.

## Introduction

The central vein sign (CVS) is currently a proposed radiological biomarker anticipated to be a criterion of McDonald 2024 in multiple sclerosis (MS) diagnoses [1, 2]. The CVS is a sensitive and specific radiological marker that distinguishes MS from other less typical demyelinating disorders or white matter (WM) lesions related to aging or cerebral small vessel disease. The perivenular course of a WM lesion could suggest the potential demyelinating nature of the WM lesion as this might play a key role in recognizing the early MS or radiologically isolated syndrome (RIS) [2, 3]. Susceptibility-weighted imaging (SWI) was initially introduced as a method to visualize the perivenular course of WM lesions [4, 5]. Currently, advanced MRI acquisition techniques with parallel imaging methods enable acquiring susceptibility signal with 3D acquisition with less than one mm voxel dimensions. Hence, 3D-isotropic acquisition of segmented Epi-T2* (Echo planar imaging T2 star) was recommended as the best achievable method for identifying the CVS [6, 7]. The spatial fusion of 3D Epi-T2* with 3D FLAIR enhances the conspicuity of WM lesion margins and improves visualization of the CVS by combining WM lesion-sensitive (via FLAIR) and vein-sensitive (via Epi-T2*) contrasts. This approach was initially demonstrated by Sati et al. through the development of the FLAIR-T2* fusion technique [8]. Subsequent studies confirmed that such fusion imaging improves the diagnostic yield of CVS by better delineating the anatomical relationship between the central vein and WM lesion [3, 9].

The majority of current cohorts reported the CVS prevalence in MS or other less typical demyelinating disorders in 3 T or 7 T magnetic field strength, as the magnitude of susceptibility rises higher compared to lower magnetic field strength. Thus, the contemporary CVS analysis methods like select-n* or percentage-based CVS+ ratio were proposed in 3 T and 7 T magnetic field strengths [9–11]. Occasionally, the performance of the 1.5 T magnetic field strength was investigated, and a few studies reported promising outcomes from Epi-T2* in identifying the CVS, despite the lower susceptibility sensitivity of Epi-T2* at 1.5 T magnetic field strength. [12, 13]. A pooled meta-analysis reported that the proportion of CVS-positive lesions in studies with 1.5 T MRI scanners was approximately 58%, whereas the corresponding pooled prevalence at 3 T was around 74% among patients with MS. Notably in this meta-analysis, the 1.5 T MRI data predominantly originated from studies conducted with susceptibility-weighted imaging (SWI), while the majority of 3 T investigations were performed with isotropic Epi-T2* [9]. Given this methodological disparity, the diagnostic utility of isotropic Epi-T2* imaging in combination with isotropic FLAIR at 1.5 T MRI warrants further evaluation to determine its feasibility for reliable CVS detection.

1.5 T MRI scanners remain widely utilized in clinical practice worldwide, both for assisting in the diagnosis of multiple sclerosis (MS) and for surveillance imaging in demyelinating diseases. The central vein sign (CVS) has the potential to serve as a practical biomarker in the diagnostic evaluation of MS. In this prospective observational study, our primary objective is to evaluate the performance of CVS assessment using isotropic 1.5 T FLAIR-T2* fusion imaging with an established select-n* and percentage-based CVS ratio method. Additionally, we aim to compare the performance of 1.5 T FLAIR-T2* fusion with that of 3 T MRI counterpart in the same cohort of patients with MS to assess the feasibility of CVS detection at 1.5 T magnetic field strength.

## Material & Method

### Study Participant

This prospective observational study was conducted at a single center from March 2023 to March 2024. The Local Clinical Observational Research Committee approved the Institutional Review Board. Sample size determination was calculated with power analysis using prior multicenter study outcomes, which reported an area under the curve (AUC) of 0.90 for a 43% CVS+ cut-off in distinguishing the MS patients from control subjects, with sensitivity of 92% and specificity of 80% [11]. Using the desired power of 90% (β = 0.10), the analysis determined that a minimum of 16 MS and 16 control subjects were required for the comparison. Our study included 20 patients diagnosed with multiple sclerosis (MS) according to the revised 2017 McDonald criteria. All patients were over 18 years old and undergoing regular surveillance without a history of major cardiovascular events. Additionally, none had significant cardiovascular comorbidities, such as diabetes mellitus, hypertension, or dyslipidemia. Out of 20 participants, six patients were active or ex-smokers.

The control subjects consisted of 20 individuals who underwent brain MRI and had no history or clinical signs suggestive of a demyelinating disorder. The majority of control subjects were referred to neurology outpatient clinics for chronic headaches, migraines (with or without aura), or suspected transient ischemic attacks (TIA). At first, potential candidates were retrospectively identified using the picture archiving and communication system (PACS), focusing on axial 2D-FLAIR and axial T2-weighted (T2W) images. During the candidate selection process, care was taken by having multiple scattered and distinct WM lesions likely of vascular origin, presumed to represent cerebral small vessel disease or migraine-related WM abnormalities that have been reported as potential mimickers of a demyelinating disorder [10]. Each control subject was re-evaluated by the referring physician (coauthor, A.A.) to confirm the absence of clinical features indicative of MS or other atypical demyelinating disorders, including MOG antibody-associated disease (MOGAD) and neuromyelitis optica spectrum disorder (NMOSD). Furthermore, potential alternative diagnoses such as Neuro-Behçet’s disease, neuro-Sjögren’s syndrome, adult-onset leukodystrophies, systemic connective tissue disorders, and hereditary vasculopathies were systematically excluded during the selection process. Verification was based on a thorough reassessment of clinical documentation and medical histories. Table 1 provides an overview of the clinical characteristics and associated comorbidities of the control subjects, while detailed individual profiles are presented in the Online Supplemental Data *(OSD).* Twenty appropriate control subjects were scanned with isotropic Epi-T2* and isotropic FLAIR in both 1.5 T and 3 T scanners. Written informed consent was obtained from each participant with MS and the enrolled control subjects.

**Table 1:** Clinical diagnosis and comorbidities of the control subjects

Primary diagnosis, *n* = 20	Age, median, [IQR]	Hypertension, *n*	Hyperlipidemia, *n*	Diabetes Mellitus, n	Smoking, *n*
Migraine with aura, *n* = 3	34 [16]	1	None	None	1
Migraine without aura, *n* = 5	28 [10]	None	None	None	2
Other chronic headache types, *n* = 4	38 [19]	None	1	None	2
Small vessel vascular disease, *n* = 8	57 [20]	5	5	4	4

*IQR* interquartile range

### MRI Acquisition Parameters and Postprocessing Techniques

3D FLAIR and 3D-segmented Epi-T2* were obtained with 0.8 mm isotropic resolution with the same axial FOV both in 1.5 T and 3 T scanners. A 16-channel parallel imaging receiver head coil was utilized on the 1.5 T (Aera, Siemens, Germany) and 3 T (Skyra, Siemens, Germany) scanners. Image acquisition parameters are listed in Table 2. Unlike the original FLAIR* technique described by Sati et al., which involves voxel-wise multiplication of FLAIR and T2* images, the approach used in this study employed a different fusion method. The original technique also requires additional postprocessing steps, including skull and dural stripping to reduce inhomogenities along the cerebral convexities [8]. In contrast, isotropic FLAIR-T2* fusion images in this cohort were created by averaging voxel signal intensities from two sources, rather than using a multiplication approach. Specifically, the fusion was achieved by weighting the voxel signal intensities with 25% derived from isotropic FLAIR and 75% from isotropic Epi-T2*. This straightforward averaging approach allows for easy postprocessing while preserving venous contrast and maintaining clear margins of WM lesions. The resulting fusion series generated at 1.5 T, using 25% signal contribution from isotropic FLAIR and 75% from isotropic Epi-T2*, is referred to as the 1.5 T FLAIR-T2* fusion series. Similarly, the final fusion series obtained from the 3 T acquisitions using the same weighted signal combination is defined as the 3 T FLAIR-T2* fusion series. These naming conventions are used consistently to denote the final post-processed fusion images specific to each magnetic field strength. The coregistration process was carried out in the Syngo.via VB40 XA (Siemens Healthcare, Erlangen, Germany) software. Both sagittal 3D-FLAIR and segmented T2-Epi* were merged with the aid of the software by the semiautomatic method. Final isotropic FLAIR-T2* fusion images were examined in terms of distortions or wrapping due to the non-local inhomogeneities along the cerebral convexities. Any minor wrapping artifact, particularly in the frontal regions, was carefully reviewed to confirm diagnostic interpretability of the final isotropic FLAIR-T2* fusion series. The duration of the semiautomatic spatial coregistration procedure was approximately four minutes. The sagittal isotropic FLAIR-T2* fusion images were also reformatted into axial and coronal planes. Axial or coronal isotropic Epi-T2* acquisitions were not preferred to avoid artifacts caused by susceptibility variations from inferior anatomical structures adjacent to the field of view, such as the neck or shoulder.

**Table 2:** MRI acquisition parameters

Sequences	1.5 T T2* EPI	1.5 T FLAIR	3 T T2* EPI	3 T FLAIR
Time (minute)	4:02	4:50	4:10	4:21
TR/TE (ms)	64/37	6000/397	55/11	7000/382
FA (degrees)	10	–	10	–
EPI factor	15	–	9	–
Turbo factor	–	220	–	250
Orientation	Sagittal	Sagittal	Sagittal	Sagittal
Bandwidth (Hz per pixel)	424 Hz	651 Hz	752 Hz	643 Hz
Acquisition voxel size (mm)	0.8 × 0.8 × 0.8	0.8 × 0.8 × 0.8	0.8 × 0.8 × 0.8	0.8 × 0.8 × 0.8
Axial FOV (mm)	244 × 244	244 × 244	230 × 230	230 × 230

*EPI* echo-planar imaging; *FLAIR* fluid-attenuated inversion recovery; *min* minutes; *TR* time to repeat; *TE* time to echo; *mm* millimeters; *ms* milliseconds; *FA* flip angle; *FOV* field of view

### Image Analysis

Each participant scanned with 1.5 T and 3 T FLAIR-T2* fusion underwent CVS assessment with a minimum two-week gap between scans to prevent recall bias. Each case was independently evaluated by two observers: a central reader, a fellowship-trained neuroradiologist with over five years of experience (Y.E.S.), and a co-reader, a board-certified radiologist in neuroradiology (A.P.) with five years of experience. To assess intrareader reliability, 10 subsets from both the MS group and the control subjects were re-evaluated four weeks after the initial assessment for both 1.5 T and 3 T isotropic FLAIR-T2* fusion.

Lesions were classified by location into four categories: subcortical, deep, periventricular, and infratentorial WM. Subcortical WM lesions were classified if the lesion was located within a 5 mm range from the adjacent cortex. The juxtacortical WM lesion contacting the cerebral cortex is evaluated within the subcortical WM lesion category. Deep WM lesions were defined as WM lesions located more than 5 mm away from the neighboring cortex without contacting the ventricular margins. The WM lesions in the brain stem or cerebellum were classified as infratentorial WM lesions. The NAIMS criteria were applied to determine the eligibility of WM lesions for CVS assessment, requiring that each lesion measure at least 3 mm in all dimensions and show no evidence of coalescence [6].

### CVS Analysis Methods

The CVS is considered positive in a non-coalescent white matter lesion when the T2*-hypointense line appears as a thin hypointense line or a small central dot. These features must be identified centrally within the WM lesion and visible at least in two orthogonal MRI planes, typically in axial, coronal, and sagittal planes [6].

The CVS analysis was performed with select-n* and a percentage-based CVS ratio for each participant. For the select-n*, the most recognized Select-3* and Select-6* are utilized. In the Select-3* approach, a case is classified positive if at least three white matter (WM) lesions are CVS+ in the deep, subcortical, or juxtacortical WM, irrespective of the total eligible lesion number. The periventricular WM lesions are excluded from the CVS analysis in the select-3* method [14]. This method offers simplicity due to its ease of implementation.

The Select-6* method is considered positive when six or more CVS+ WM lesions are identified, irrespective of WM lesion location, in contrast to Select-3*. When the total number of CVS-eligible lesions is less than six, in that case, the Select-6* is considered positive only if the number of CVS-positive WM lesions is greater than the number of CVS-negative WM lesions. [7, 15]. This conditional rule is proposed to use the Select-6* system in subjects with a limited eligible WM lesion.

The percentage-based CVS analysis was performed by calculating the proportion of CVS+ lesions among total CVS-eligible lesions for each subject. For this purpose, various threshold levels, such as 30%, 40%, and 50%, were considered to support the diagnosis of MS over non-MS conditions, including small vessel ischemic changes, migraine, and other less typical demyelinating disorders. Among these thresholds, the 40% CVS+ cut-off achieved the highest accuracy based on the largest comprehensive meta-analysis conducted by Castellaro et al. [9]. Accordingly, a 40% cut-off was adopted in our analysis to assess the diagnostic performance of both 1.5 T and 3 T FLAIR-T2* fusion imaging.

### Statistical Analysis

All statistical analyses were computed by SPSS-28 (IBM SPSS Statistics, Armonk, NY, USA). The normality analysis was performed with the Shapiro-Wilk test for each variable. We referred to any outcomes from the 1.5 T and 3 T isotropic FLAIR-T2* as dependent variables. Independent sample t‑tests were used to seek the difference in the CVS+ ratio of MS patients from control subjects in both magnetic field strengths. The paired t‑test was utilized to compare the CVS+ ratio between the isotropic FLAIR-T2* with 1.5 T and 3 T in the participants with MS. The non-parametric continuous variables, like the CVS+ ratio of subcortical, deep, periventricular, or infratentorial WM lesions were compared by the Wilcoxon signed rank test among both scanners. The Pearson chi-square test was used for the comparison of the select-n* system in both scanners. The sensitivity and specificity of select-n* outcomes are also measured.

To assess the consistency of the outcome, an additional sensitivity analysis was performed by randomly selecting two lesions—one from the periventricular white matter and one from the deep white matter—for each MS participant and the control subjects. The CVS+ proportions of these randomly selected WM lesions were compared to the overall CVS+ proportions derived from the full dataset across all participants in both 1.5 T and 3 T MRI scans. A two-proportion Z‑test was performed, along with 95% confidence intervals (CIs) were calculated to assess the consistency of the CVS+ proportions.

To assess the accuracy of 1.5 T and 3 T isotropic FLAIR-T2*, the receiver operating characteristics (ROC) curve was generated by computing the normally distributed CVS+ ratio of MS patients in both scanners, with the addition of CVS+ proportions from the control subjects. The best cut-off values were selected based on the highest Youden index from the point on the ROC curves. Additionally, the sensitivity and specificity of each test were measured by accounting for the pre-determined CVS threshold of 40% for each magnetic field strength. Cohen’s Kappa was used to assess intra- and inter-reader reliability analysis. A *p*-value of less than 0.05 was used as the significance threshold for all statistical analyses.

## Results

### Clinical Characteristics

Among 20 MS patients, a total of 258 WM lesions were identified as eligible for CVS assessment in both 3D-FLAIR of 1.5 T and 3 T magnetic field strengths. Two hundred fifty-five WM lesions were eligible for the CVS assessment among the 20 control subjects. The clinical characteristics and demographics are presented in Table 3.

**Table 3:** Distribution of study participants based on demographics and clinical features

Variables	MS group, *n*:20	Control subjects, *n*:20	*p*-value
Age, year (mean ± SD)	33.5 ± 3.1	40 ± 4.3	0.01
Sex, (M/F)	10/10	10/10	–
EDSS Score	2.3 ± 1.4	–	–
Oligoclonal band positive, *n*, (%)	18, (90)	–	–
Disease duration*, (years), median, [IQR]	5, [9]	–	–

*MS* Multiple Sclerosis; *EDSS* Extended Disability Status Scale; *IQR* Interquartile Range

*Time from disease onset to the study period

### CVS Status of WM Lesions in Patients with MS and Control Subjects in Different MRI Field Strengths

**Fig. 1:**
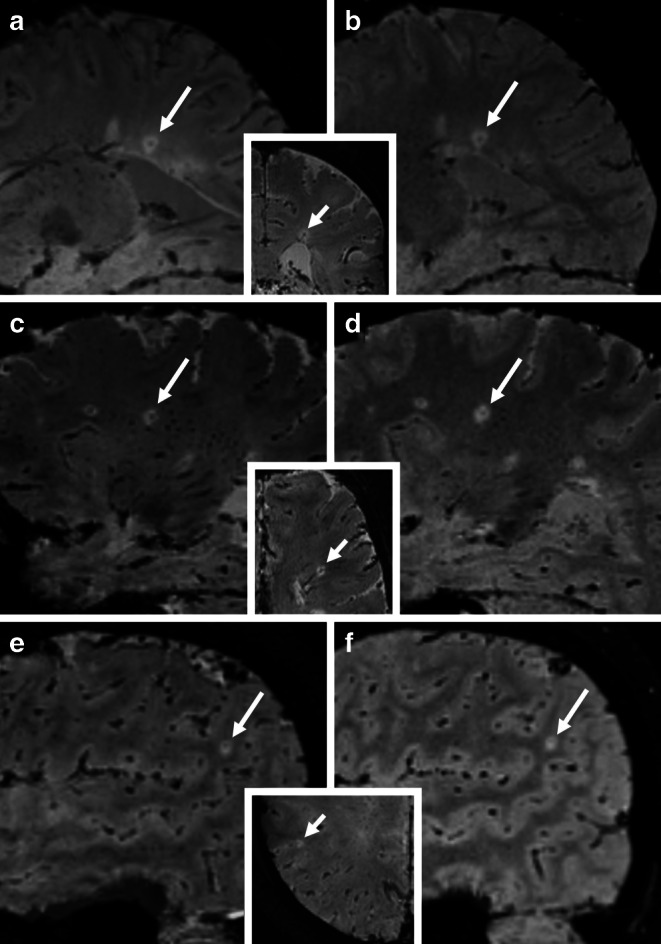
Illustration of the isotropic FLAIR-T2* fusion imaging in the central vein sign (CVS) assessment of multiple sclerosis (MS) subjects at 1.5 T and 3 T magnetic field strength. **a**, **b** Sagittal isotropic FLAIR-T2* shows a CVS+ periventricular lesion from the participant with MS (arrows) with (**a**) depicting the 3 T FLAIR-T2* and (**b**) illustrating the 1.5 T FLAIR-T2* fusion imagings. The middle image demonstrates a coronal view confirming the periventricular location. **c**, **d** A CVS+ demyelinating lesion in deep white matter (arrows) is noted in a participant with MS, with (**c**) depicting the 3 T FLAIR-T2* and (**d**) illustrating the 1.5 T FLAIR-T2* fusion imaging. The middle image provides an axial view of the lesion’s deep white matter location. Both raters classified this lesion as CVS+ on 1.5 T FLAIR-T2* fusion despite being less distinct than 3 T FLAIR-T2* fusion. **e**, **f** Sagittal FLAIR-T2* fusion illustrates a subcortical CVS+ demyelinating lesion (arrows) in a participants with MS, with (**e**) from 3 T MRI and (**f**) from 1.5 T MRI. The middle image with an axial view confirms the subcortical localization. Despite being less conspicuous on 1.5 T FLAIR-T2* fusion, this lesion was classified as CVS+ by both raters

**Fig. 2:**
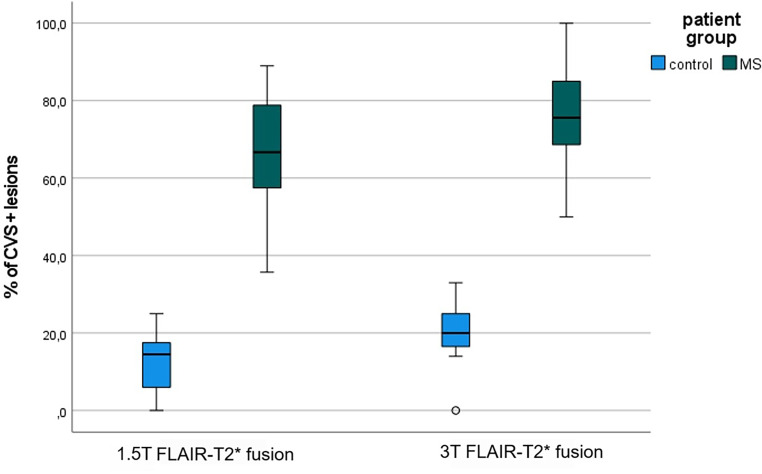
Plot-box illustration of the CVS+ ratio in white matter lesions based on percentage-based method. Multiple sclerosis patients showed a significantly higher proportion of CVS+ lesions than the control subjects in both 1.5 T and 3 T isotropic FLAIR-T2* fusion imaging (*p* < 0.01). Furthermore, 3 T FLAIR-T2* fusion demonstrated a greater CVS+ proportion than 1.5 T FLAIR-T2* fusion in MS participants. At the same time, no significant difference was observed in the CVS proportion of the control subjects between both magnetic field strengths (*p* = 0.13)

Twenty MS patients revealed a mean of 66.9 ± 15.4% CVS+ lesions per participant in the 1.5 T FLAIR-T2* fusion group and 77.0 ± 13.6% CVS+ lesions per participant in the 3 T FLAIR-T2* fusion group (*p* < 0.01). Fig. 1 illustrates the CVS+ WM lesions detected in 1.5 T and 3 T FLAIR-T2* fusion groups, respectively. The control subjects revealed a mean of 12.6 ± 8.3% CVS+ lesions in 1.5 T FLAIR-T2* fusion, and 19.4 ± 10.4% CVS+ lesions in 3 T FLAIR-T2* fusion imaging (*p* = 0.13). Plot boxes are presented for both techniques in Fig. 2**.**

The sensitivity analysis confirmed the consistency of CVS+ proportion outcomes across both 1.5 T and 3 T magnetic field strengths. As detailed in Table 4, the CVS proportions remained largely stable in all participants with MS, with a statistically insignificant increase in the randomly selected WM lesion subsets in both magnetic field strengths (*p* = 0.12 at 1.5 T FLAIR-T2* and *p* = 0.06 at 3 T FLAIR-T2* fusion). These findings suggested that the CVS+ proportion outcomes are not excessively inflated and remain robust across either 1.5 T or 3 T scanners. In the control subjects, the CVS proportions were nearly identical between the entire dataset and the randomly selected WM lesions, further supporting the consistency of the results.

**Table 4:** Overall CVS+ proportions of MS participants and the control subjects, including sensitivity analysis with confidence intervals

Group	Dataset	1.5 T CVS+ ratio and 95% CI	3T CVS+ ratio and 95% CI	Z*-value (1.5 T)	*p*-value (1.5 T)	Z*-value (3 T)	*p*-value (3 T)
MS participants	Main Dataset	0.62, [0.56, 0.68]	0.73, [0.67, 0.78]	−1.57	0.12	−1.98	0.06
	Randomly selected lesions	0.75, [0.62, 0.88]	0.88, [0.77, 0.98]				
Control subjects	Main Dataset	0.17, [0.13, 0.22]	0.17, [0.13, 0.22]	−0.04	0.97	−0.04	0.97
	Randomly selected lesions	0.18, [0.06, 0.29]	0.18, [0.06, 0.29]				

Abbreviations: *CVS* Central vein sign; *CI* Confidence interval; *MS* Multiple Sclerosis

*Two-proportion Z‑test

### Accuracy of 1.5 T and 3 T Isotropic FLAIR-T2* Fusion in CVS Assessment

**Fig. 3:**
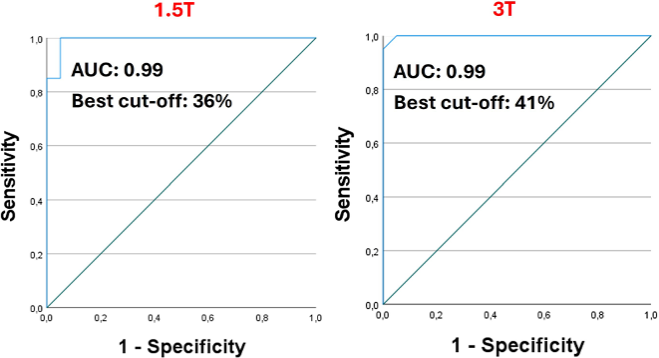
Receiver operating characteristic (ROC) curves for CVS+ proportion in 1.5 T and 3 T FLAIR-T2* fusion Imaging. In the current study, 1.5 T FLAIR-T2* fusion imaging achieved 92.5% accuracy, with 90% sensitivity and 95% specificity using a 40% CVS threshold system. Similarly, 3 T FLAIR-T2* fusion imaging achieved 97.5% accuracy, with 100% sensitivity and 95% specificity with the same cut-off. Based on the best Youden indexes, the optimal discriminative CVS proportion cut-off was 36% (sensitivity = 95%, specificity = 95%) for 1.5 T FLAIR-T2* fusion imaging and 41% (sensitivity = 100%, specificity = 95%) for 3 T FLAIR-T2* fusion imaging

The diagnostic performance of 1.5 T and 3 T FLAIR-T2* fusion imaging in distinguishing MS patients from control subjects was comparable. ROC curve analysis demonstrated near-perfect accuracy for both 1.5 T and 3 T FLAIR-T2* fusion in distinguishing MS patients from control subjects, as presented in Fig. 3. The 1.5 T FLAIR-T2* fusion achieved a sensitivity of 90% and a specificity of 95% using a 40% threshold system, while the 3 T FLAIR-T2* fusion yielded a sensitivity of 100% and a specificity of 95% with the same 40% cut-off. Based on the highest Youden index, the optimal cut-off derived from the ROC curve was 36% for 1.5 T FLAIR-T2* and 41% for 3 T FLAIR-T2* fusion imaging to distinguish MS participants from control subjects.

### Select-n* Performance in Different Magnetic Field Strengths

Select-3* demonstrated limited specificity across both magnetic field strengths, yet maintained high sensitivity (95%) in distinguishing MS patients from control subjects. On the other hand, the Select-6* system achieved 95% sensitivity and specificity in both 1.5 T and 3 T FLAIR-T2* fusion imaging. select-n* system performance is listed in Table 5.

**Table 5:** Performance of Select-n* system in both magnetic field strengths for MS diagnosis

Select-n* system, *n* = 40		Sensitivity (%)	Specificity (%)
1.5 T isotropic FLAIR-T2* fusion	Select-3*	95	65
	Select-6*	95	95
3T isotropic FLAIR-T2* fusion	Select-3*	95	70
	Select-6*	95	95

*MS* Multiple Sclerosis; *FLAIR* fluid-attenuated inversion recovery

### Location-based CVS+ Proportions

Among the 258 CVS-eligible WM lesions in MS participants, the periventricular region (*n* = 101) was the most frequent WM location. On the contrary, the deep WM lesions were the most common location in the control group (*n* = 163). 1.5 T FLAIR-T2* fusion was less effective in identifying CVS+ lesions in the deep WM compared to 3 T FLAIR-T2* fusion, with median ratios of 57.1 and 87.5%, respectively, (*p* = 0.05). There was no significant difference in the CVS+ proportion between 1.5 T and 3 T FLAIR-T2* fusion for the periventricular, subcortical, and infratentorial WM lesions, although subcortical and infratentorial WM lesions were not common in participants with MS in the current study. Location-based stratification of CVS+ lesions is presented in Table 6.

**Table 6:** CVS+ outcomes in MS patients stratified by location in different magnetic field strengths

(Location and total number of CVS-eligible lesions, *n*)	1.5 T FLAIR-T2* fusion CVS+ (%), median [IQR]	3T FLAIR-T2* fusion CVS+ (%), median [IQR]	*p*
Periventricular WM, *n* = 101	70.0 [13.3]	83.3, [40]	0.13^a^
Deep WM, *n* = 82	57.1 [50.0]	87.5, [33.3]	0.05^a^
Subcortical WM, *n* = 58	50.0 [60.0]	80.0, [40.0]	0.23^a^
Infratentorial WM, *n* = 17	66.7 [100.0]	90.0, [100]	0.47^a^

*WM* white matter; *CVS* central vein sign; *IQR* interquartile range; *FLAIR* fluid-attenuated inversion recovery; *MS* multiple sclerosis

^a^Wilcoxon Signed Rank test

### Interrater and Intra-rater Reliability

In WM lesion-based CVS analysis of participants with MS, interrater agreement between the central and co-rater was substantial (ҡ: 0.77) in 1.5 T FLAIR-T2* and near perfect (ҡ: 0.88) in 3 T FLAIR-T2* fusion imaging. In WM lesion-based CVS analysis of the control subjects, interrater agreement between the central and co-rater was substantial (ҡ: 0.72) in 1.5 T FLAIR-T2* and good (ҡ: 0.81) in 3 T FLAIR-T2* fusion imaging. Intra-rater agreement in participants with MS was substantial (ҡ: 0.80) in 1.5 T FLAIR-T2* and near-perfect (ҡ: 0.92) in 3 T FLAIR-T2* fusion imaging.

## Discussion

The central vein sign (CVS) is a recently recognized biomarker for differentiating multiple sclerosis (MS) or clinically isolated syndrome (CIS) from non-MS inflammatory disorders or cerebral small vessel disease [11, 16]. In recent decades, numerous studies have attempted to visualize perivenular demyelinating lesions using susceptibility-based techniques like SWI [4, 5]. However, 3D EPI-T2* has now demonstrated superior performance in revealing the central course of the vein within lesions. Most studies about CVS are performed with 3 T scanners, although several reports highlight the high performance of 1.5 T in depicting the CVS with 3D Epi-T2* [3, 13]. The current study compares the performance of 1.5 T and 3 T Epi-T2* combined with FLAIR fusion imaging in the same group of MS participants and control subjects. These findings indicate that Epi-T2* imaging at 1.5 T may provide diagnostic performance comparable to 3 T for CVS detection, effectively distinguishing participants with MS from the WM lesion of nondemyelinating processes such as migraine or small vessel ischemic gliotic changes.

Previous studies performed with a 1.5 T magnetic field strength have predominantly used SWI and reported a variably high frequency of CVS+ WM lesions. They suggested the promising efficiency of the 1.5 T magnetic field strength in discriminating MS from cerebral small vessel vasculopathy [12, 17, 18]. Reported CVS+ ratios in MS patients range from 30 to 50%, while cerebral small vessel disease lesions revealed CVS+ proportions between 10 and 30%. In a multicenter series, Maggi et al. reported median CVS+ ratios of 88% with 1.5 T isotropic Epi-T2* and 85% with 3 T isotropic Epi-T2* imaging in MS patients, although the participants were different between both scanners [3]. Due to the limited number of studies on 3D Epi-T2*-based CVS assessment at 1.5 T, the widely recognized 40% cut-off for MS/CIS diagnosis is primarily based on data from 3 T and 7 T Epi-T2* series [9, 15, 17, 19]. Our analysis achieved the optimal cut-off of 36% in 1.5 T FLAIR-T2* and 41% in 3 T FLAIR-T2* fusion in the same patient group with high accuracy. This difference is attributed to the low study population and a greater likelihood of classifying CVS+ for an MS participant in isotropic FLAIR-T2* fusion imaging at 3 T MRI. Two different meta-analyses suggest that 3 T outperforms 1.5 T in detecting CVS, with proposed cut-offs of 40 and 45%, respectively [9, 20]. However, the majority of CVS identification in 1.5 T MRI in these meta-analyses was performed with SWI, suggesting that the isotropic Epi-T2* technique at both 1.5 T and 3 T may enhance visualization of the perivenular course of WM lesions and reduce field strength-related variability among both scanners. Our preliminary findings may support the hypothesis by showing the feasibility of 3D Epi-T2* applications in a 1.5 T magnetic field strength with a 40% CVS+ recommended cut-off, although the diagnostic performance remained marginally lower than that observed at 3 T magnetic field strength.

The location-based distribution of WM lesions revealed a higher prevalence of CVS in deep WM lesions in 3 T FLAIR-T2* than in its 1.5 T counterpart. At the same time, no statistically significant differences were observed in the distribution of CVS in other WM locations. A key limitation of this finding is the uneven distribution of WM lesions across the four different WM compartments, particularly the lower number of infratentorial WM lesions. Regardless, CVS+ deep WM lesions were more commonly identified on 3 T FLAIR-T2* fusion with mild statistical significance. The discrepancy in the characterization of deep WM lesions may be attributed to ill-defined T2 hypointense signals observed on 1.5 T FLAIR-T2 fusion, which are more conspicuously visualized as CVS-positive at 3 T magnetic field strength. Tallantyre et al. and Hosseini et al. reported that deep WM lesions in RRMS tend to show fewer CVS+ lesions compared to other regions [19, 21]. Detection of CVS-positive lesions in deep WM is particularly important in patients with a low lesion burden or in those lacking WM lesions in typical locations associated with primary demyelination, such as the periventricular, infratentorial, or juxtacortical regions [22]. In such cases, differentiation between the demyelinating lesion and those of small vessel ischemic lesion on 1.5 T FLAIR-T2* fusion may be less effective than 3 T FLAIR-T2* fusion, particularly for small lesions (< 3 mm in any size) where even the 3 T magnetic field strength might not provide high sensitivity like isotropic Epi-T2* of 7 T as reported by Okromelidze et al. [10]. However, validation in larger cohorts is warranted to reveal the location-based differences in the frequency of CVS-positive WM lesions across the different magnetic field strengths.

Our study has several limitations. First, our cohort consisted exclusively of MS patients with long-standing disease, lacking presentation of other less typical conditions such as autoimmune demyelinating disorders or inflammatory vasculopathy. In these cases, larger studies have reported a mean perivenular WM lesion ratio of 20–30% [2, 3]. Including these atypical presentations could provide a more comprehensive understanding of the role of the CVS sign, particularly when using low-field isotropic Epi-T2* imaging. Distinguishing the MS/CIS from these disorders can sometimes be challenging in routine clinical practice with standard brain MRI. Secondly, the small sample size also introduces heterogeneity, particularly in the percentage-based CVS+ proportion measurement at the patient level, as including both high and low lesion load MS cases may result in a close CVS+ proportion. However, the sensitivity analysis proved no abnormal inflation in the CVS+ proportions, and the required sample size determination was made based on outcomes from a prior multicenter study. The relatively limited number of participants may still introduce vulnerability in calculated diagnostic performance metrics of FLAIR-T2* in both scanners, such as sensitivity, specificity, and accuracy values. Therefore, possible variations should be kept in mind when interpreting results from a relatively small dataset, and large-scale prospective studies are needed to validate these preliminary findings. Nevertheless, select-n* methods might be more practical and could reduce lesion load-related heterogeneity, as the performance of Select-3* and Select-6* methods was not different across 1.5 T and 3 T FLAIR-T2* fusion in our series. Additionally, the active demyelination status of each WM lesion in the MS group was not evaluated during the CVS assessment. Active demyelination often causes lesions to enlarge or coalesce, potentially involving multiple central veins and precluding the WM lesion from CVS assessment. However, none of the patients in our series showed an objective clinical attack. Nonetheless, the current understanding of chronic active demyelination in clinically silent cases should be considered during CVS assessment, as these lesions tend to degenerate centrally and gradually increase in size, which might potentially lead to underestimating the CVS proportion in MS patients.

## Conclusion

Isotropic FLAIR-T2* fusion imaging at 1.5 T may demonstrate efficient and reliable CVS assessment, although outperformed by 3 T FLAIR-T2* fusion based on a percentage-based method. These preliminary findings suggest that CVS analysis using isotropic Epi-T2* imaging is also feasible at 1.5 T magnetic field strength and may support MS diagnosis in settings where a 3 T scanner is not available. This initial finding holds significance, considering the widespread use of 1.5 T magnetic field systems globally.

## Supplementary Information

Clinical and Demographic Features of Control Subjects
