# Supplementary material for: Diagnostic Accuracy of Isotropic FLAIR-T2* Fusion Imaging for Central Vein Sign Detection in Multiple Sclerosis: a Comparative Study at 1.5 T and 3 T
Source: Clin Neuroradiol. 2025 Jun 16;35(4):725–33. doi: 10.1007/s00062-025-01531-6 (PMC12552248; doi:10.1007/s00062-025-01531-6)
Supplement: Supplementary file 1 — Clinical and Demographic Features of Control Subjects [file 62_2025_1531_MOESM1_ESM.docx]

# Clinical and Demographic Features of Control Subjects

| ControlSubject | Primary Diagnosis | Onset Symptom | Hypertension | Dyslipidemia | Diabetes Mellitus | Smoking Status | History of Stroke/Thromboembolism |
| --- | --- | --- | --- | --- | --- | --- | --- |
| C01 | Migraine with aura | Left hemifacial headache | None | None | None | Never | None |
| C02 | Migraine without aura | Headache | None | None | None | Never | None |
| C03 | Migraine with aura | Periorbital numbness with Headache | None | None | None | Never | None |
| C04 | Migraine without aura | Headache, photophobia | None | None | None | Ex-smoker | None |
| C05 | Migraine without aura | Headache Numbness | None | None | None | Ex-smoker | None |
| C06 | Migraine with aura | Visual disturbance, headache, hemihypoesthesia | Systolic hypertension | None | None | Current smoker | None |
| C07 | Migraine without aura | Headache | None | None | None | Never | None |
| C08 | Migraine without aura | Headache | None | None | None | Never | None |
| C09 | Tension-type headache | Headache | None | None | None | Ex-smoker | None |
| C10 | Tension-type headache | Headache | None | None | None | Never | None |
| C11 | Tension-type headache | Headache | None | None | None | Never | None |
| C12 | Tension-type headache | Headache | None | None | None | Current | None |
| C13 | Small vessel vascular disease | Perioral numbness | Systolic-diastolic hypertension | Hypercholestrolemia | None | Current | None |
| C14 | Small vessel vascular disease | Screening | None | None | Type 2 DM | Never | Prior transient ischemic attack |
| C15 | Small vessel vascular disease | Screening | Systolic-diastolic hypertension | Hypercholestrolemia | Type 2 DM | Never | None |
| C16 | Small vessel vascular disease | Screening | None | None | Type 2 DM | Current | None |
| C17 | Small vessel vascular disease | Vertigo | None | Hypercholestrolemia | Type 2 DM | Current | None |
| C18 | Small vessel vascular disease | Lightheadness | Systolic hypertension | Hypercholestrolemia | None | Never | None |
| C19 | Small vessel vascular disease | Left upper extremity numbness | Systolic hypertension | Hypercholestrolemia | None | Never | None |
| C20 | Small vessel vascular disease | Amorozis fugax | Diastolic Hypertension | No | Type 2 DM | Current | Prior transient ischemic attack |
